# Investigation of bacterial diversity using 16S rRNA sequencing and prediction of its functionalities in Moroccan phosphate mine ecosystem

**DOI:** 10.1038/s41598-022-07765-5

**Published:** 2022-03-08

**Authors:** Salah Eddine Azaroual, Yassine Kasmi, Abderrahim Aasfar, Hicham El Arroussi, Youssef Zeroual, Youssef El Kadiri, Abdelali Zrhidri, Elmostafa Elfahime, Abdelaziz Sefiani, Issam Meftah Kadmiri

**Affiliations:** 1 Green Biotechnology Laboratory, Moroccan Foundation for Advanced Science, Innovation and Research (MAScIR), Rabat Design Centre, Rue Mohamed Al Jazouli, Madinate Al Irfane, 10100 Rabat, Morocco; 2 Mohammadia School of Engineering, Process Engineering Research Team, Mohammed V University, Rabat, Morocco; 3 Situation Innovation Group–OCP Group, Jorf Lasfar, Morocco; 4 Department of Medical Genetics, National Institute of Health, Rabat, Morocco; 5 Research Team in Genomics and Molecular Epidemiology of Genetic Diseases, Genomic Center of Human Pathologies, Faculty of Medicine and Pharmacy, Mohammed V University in Rabat, Rabat, Morocco; 6 National Center for Scientific and Technical Research/Rabat (CNRST), Angle Avenue Allal El Fassi, avenue des FAR, District Hay Ryad, United Nations, BP 8027, 10102 Rabat, Morocco; 7 AgroBioSciences, Biodiversity and Plant Sciences, Plant and Soil Microbiome Sub-Program, Mohammed VI Polytechnic University, Ben Guerir, Morocco

**Keywords:** Metagenomics, Microbial communities, Soil microbiology

## Abstract

Native plants in extreme environments may harbor some unique microbial communities with particular functions to sustain their growth and tolerance to harsh conditions. The aim of this study was to investigate the bacterial communities profiles in some native plants and samples of the Moroccan phosphate mine ecosystem by assessing the percentages of taxonomic identification using six hypervariable regions of the 16S rRNA. The rhizosphere of the three wild plants in the Moroccan phosphate mine is characterized by interesting bacterial diversity including *Proteobacteria (*62.24%, 71.15% and 65.61%), *Actinobacteria* (22.53%, 15.24%, 22.30%)*, Bacteroidetes* (7.57%; 4.23%; 7.63%), and *Firmicutes* (5.82%; 1.17%; 2.83%). The bulk phosphate mine samples were dominated by *Actinobacteria* with average relative abundance of 97.73% that are different from those inferred in the rhizosphere samples of the native plants. The regions V3, V4 and V67 performed better in the taxonomic profiling at different taxonomic levels. Results indicated that both plant genotype and mainly soil conditions may be involved in the shaping of bacterial diversity. Such indication was also confirmed by the prediction of functional profiles that showed enrichment of many functions related to biological nitrogen fixation in the rhizosphere of native plants and the stress related functions in the bulk phosphate mine in comparison with the wheat rhizosphere samples.

## Introduction

Plant associated microbiota have attracted many researchers worldwide and continue to be one of the most studied research areas in microbiology over the last decades. In a recent revision and review of the concept definition and challenges in microbiome research, Berg et al.^1^ proposed an explicit definition for the term microbiome based on the previous definitions with some newly introduced amendments. The proposed definition can be summarized as the theatre of activities of microorganisms living in a giving ecosystem. According to this definition, plant microbiomes are not passive actors^2^. They can, rather, interact with host development, physiology and systematic defense (e.g., mycorrhizal symbiosis with wheat plants^3^), induce disease resistance (e.g. bacterial endophyte *Enterobacter* sp. “M6” that confers resistance to fungal pathogen *Fusarium graminearum* in Finger Millet^4^), confer fitness advantages to plant host (e.g. Association of microbiome phenotypes with Arabidopsis genes involved in immunity, cell-wall integrity, root and root hair development^5^) and increase plant tolerance to stress and drought^6–8^. In fact, plant Microbiome was identified as a key for the next green revolution^9^, and numerous products and microbiome management strategies were developed in agriculture including (i) microbiome transplants, (ii) microbial inoculants, (iii) microbial and plant extracts, (iv) methods to change environmental conditions and (V) microbiome engineering^10–12^

The analysis of the plant associated microorganisms, their composition and their activities and functions are still challenging. Traditionally, microbiological investigations of microbiota relies on the isolation and the cultivation of single isolates followed by morphological, biochemical and molecular characterizations. However, despite the continuous improvements in cultivation-dependent techniques, an often cited estimate indicated that only 1% of extant bacteria can be isolated in culture and as much as 99% or more of the microorganisms present in many natural environments are not readily culturable^13,14^. Although this traditional approach is still required in order to isolate single strains that can be applied as inoculum, the next-generation sequencing (NGS) molecular methods are, nowadays, widely used to profile microbial communities and their dynamic in different plants and plant compartments^15^. Sequencing platforms (e.g., Illumina^®^, PacBio^®^, Oxford nanopore^®^ and Ion Personal Genome Machine (PGM)^®^) have known interesting advances in wet laboratory protocols, kits and bioinformatics pipelines in plant Microbiome related applications and studies. Such advances allow cost reduction, time preservation and high accuracy in deciphering the structure and function of microbial communities in plant microbiome analysis^16,17^. The microbiome analysis with NGS platforms relies mainly on the amplicon sequencing methods that target the 16S rRNA gene^18^. Its sequences serve as a marker of choice in order to infer the microbiome composition. 16S rRNA gene is universally present in bacteria and archaebacteria, it contains conserved, variable and hypervariable regions allowing taxonomical classification and separation. These characteristics of the 16S rRNA gene allow the development of numerous primer combinations that are used to amplify hypervariable regions and to generate amplicon of variable lengths suitable for subsequent sequencing with different NGS platforms (e.g., Illumina, Oxford Nanopore, Pacific Biosciences, Ion PGM). Illumina sequencing platforms are considered as the major player among NGS platforms and the MiSeq variant is considered as the default choice for metagenomics studies^18,19^. In the illumina systems, the variable regions V3 and V4 of the 16S rRNA are predominantly sequenced and analyzed in microbial diversity studies. Similarly, Thermo scientific^TM^ developed Ion 16S^TM^ metagenomics kit that has the ability to amplify and sequence six hypervariable regions (V2, V3, V4, V6-7, V8 and V9) simultaneously^20^. However, this NGS system is rarely used in metagenomics analysis of 16S rRNA gene mainly in the context of plant–microbiome interactions. In this work we used the Ion 16S^TM^ metagenomics approach to investigate the microbial diversity in the rhizosphere and bulk soil of native plants in Moroccan phosphate mine. Barb et al.^21^ analyzed the six hypervariable regions of the 16S marker gene by using the Ion 16S^TM^ metagenomics kit to study the oral microbiome diversity. They developed a novel analytical pipeline with the V4 region enabled the best resolution of bacterial communities profiling up to both family and genus levels. In a recent study, Tamošiūnė et al.^22^ assessed the effect of cold Plasma treatment on the plant-associated Microbiome using the Ion 16S^TM^ metagenomics kit which seems to be the first study applying this kit on plant-associated microorganisms. However, in this study, authors did not give indication related to the best 16S rRNA hypervariable region that gives a robust identification and classification of taxa.

Plants are very diverse and occupy most of terrestrial ecosystems, where they play a key role as primary producers. They are also present in extreme and uncommon environments such as deserts, contaminated soils and ores. Many researchers postulated that native plants from these extreme environments may harbor some unique microbial communities with particular functions to sustain their growth and tolerance to harsh conditions^23,24^. Investigations of the microbiome of plants growing in unfavorable environments and stresses may provide insight into microbial and plant traits that allow them to alleviate such stresses (e.g., drought, salinity, pests and pathogens). In this sense, Pérez- Jaramillo et al.^25^ proposed a ‘back to the roots’ framework that comprises the exploration of the microbiomes of native plants vegetation growing in their native habitats for the identification of microbial traits to reinstate beneficial association that may have been lost during plant domestication. For examples, in Chile, the rhizospheres of *Atriplex* sp. and *Stipa* sp. (shrubs) grown in the Atacama Desert were dominated by *Gammaproteobacteria* as revealed by 454‒pyrosequencing studies^23^. Zhang et al.^24^ described and compared abundance and composition of bacterial communities associated with leaves and roots of plants grown in Atacama desert (*Distichlis spicate* (Poacea) and *Pluchea absinthioides* (Asteraceae)). Authors revealed the abundance of *Proteobacteria*, *Firmicutes*, *Actinobacteria* and *Bacteroidetes* among the endophytic bacterial communities. Interestingly, most of operational taxonomic units (OTUs) were not shared between endospheres of these indigenous plants indicating the effect of plant genotype on the bacterial communities distribution. The ability of some native isolates from Atacama desert to act as plant growth promoting bacteria (PGPB) was confirmed in wheat plants, they also showed greater protection against salt stress^26^.

Moroccan phosphate mines, mainly in the center of Morocco (Ben Guerir Location), are characterized by high temperature in the summer, aridity and rains scarcity, very low organic matter and high phosphate mineral contents. This environment can be considered as extreme and the native plants growing in this habitat could represent the optimal place to look for plant growth promoting bacteria (PGPB) with interesting and unique traits that confer resistance to crops. Unfortunately, very few microbiological studies on phosphate mines have been carried out. In one of our recent studies, we isolated and characterized bacilli strains from rock phosphate mines that showed interesting plant growth promotion traits such as phosphate solubilization, phytohormone and 1-Aminocyclopropane-1-carboxylate (ACC)-deaminase production^27^. Previously, Hamdali et al.^28,29^ studied Actinobacteria (e.g., *Streptomyces griseus* and *Micromonospora antiaca*) native in Moroccan phosphate mines that showed abilities to solubilize rock phosphate and other multiple plant growth properties under in vitro and greenhouse conditions. The above mentioned studies followed the traditional cultivation-dependent techniques, and to the best of our knowledge there is no study applying the NGS methods to describe the microbial communities in this environment.

In this work, we analyzed the effect of the six hypervariable regions in the taxonomic assignment, abundance and distribution of the bacterial communities in the studied samples. In this approach, the prediction of the bacterial functionalities based on 16S rRNA profiles was performed using Tax4fun software package available in R environment^30^. With this bioinformatics prediction, we gave insights into the functional capabilities of the microbial communities associated with natives plants in phosphate mines, in a cost-effective way in comparison with the shotgun metagenomics approach.

## Results

### Sequencing yield and pre-processing

To investigate the microbiome associated with native plants that grow in phosphate mines in Morocco and its comparison with the phosphate bulk soil samples and the rhizosphere of wheat crop, a total of 15 samples were subjected to 16S rRNA sequencing analysis using Ion Torrent PGM™ sequencing platform. Total obtained reads accounted for 2,782,897. They were filtered for chimeras, quality score and copy number. Reads were classified according to 16S rRNA variable regions for each sample and only reads with high number of copies were mapped against 16S rRNA databases (Supplementary materials Tables S1 and S2).

### Taxonomic profiling using the primers targeting different hypervariable regions of the 16S rRNA

Variable regions of the 16S rRNA were amplified in the DNA derived from the studied samples and the ZymoBIOMICS community standard using two separate primer sets with the Ion 16S™ metagenomics kit. Read Classification to taxonomic profiles at four levels e.g., phylum, family, genus and species was compared between the data of each region and the concatenated sequences of all regions according to the number of taxa identified. In this section, results obtained for the standard ZymoBIOMICS DNA were also presented.

**Figure 1:**
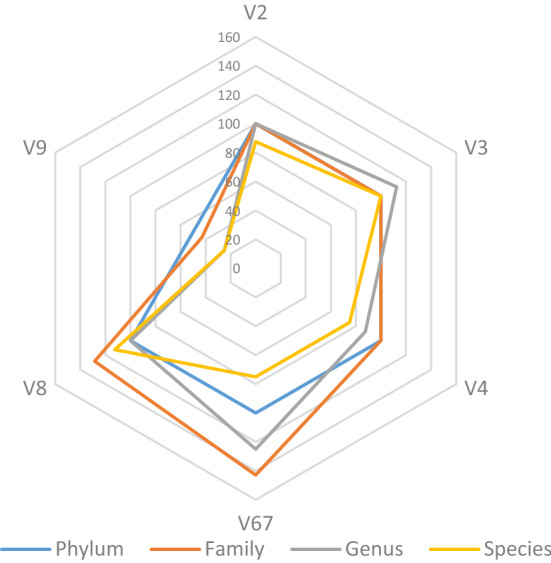
Percentages identification of Phylum, Family, Genus and species present in the ZymoBIOMICS standard according to different hypervariable regions of the 16S rRNA (V2, V3, V4, V67, V8 and V9) analyzed by the Ion 16S sequencing protocol.

Figure 1 showed that at the phylum level, V2, V3, V4, V67 and V8 performed well for the identification of the phyla present in the ZymoBIOMICS standard. However, V9 region identified only *Proteobacteria* phylum. Taxonomic profiling at more lower levels became challenging and V9 region gave less taxonomic profiling up to family, genus and species levels. V67 and V8 regions gave better taxonomic profiling than V9, nevertheless, new families and genera were found with these regions that are not present in the standard. V2, V3 and V4 regions gave the best taxonomic profiling up to both family and genus levels (Fig. 1).

**Figure 2:**
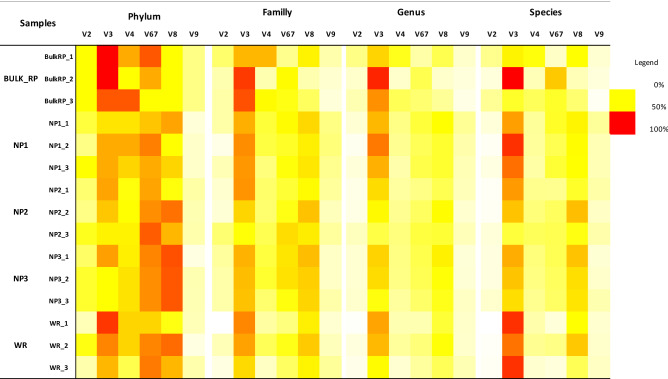
Percentages identification presented by heatmap plots of Phylum, Family, Genus and species estimated in Bulk phosphate mines (Bulk RP); Alternanthera caracasana (NP1); Digitaria sanguinalis (NP2); Dittrichia Viscosa (NP3) and wheat crop (WR) samples according to different hypervariable regions of the 16S rRNA (V2, V3, V4, V67, V8 and V9) analyzed by the Ion 16S sequencing protocol.

The same analysis was performed for the collected soil samples (Fig. 2). However, since the bacterial composition of these samples is unknown compared to the standard ZymoBIOMICS, the maximum number of each taxonomic level present in each sample was taken as the target identification percentage. For each region, taxonomic profiling percentages were calculated accordingly and presented as heatmap. The overall analysis of the Fig. 2 indicated variability in the taxonomic profiling of different 16S rRNA hypervariable regions according to the taxonomic level and the sample types. However, some regions gave the lesser identification percentages e.g., V9 and V2 whereas V3, V4, V67 and V8 performed better in the taxonomic profiling mainly at the phylum level. At lower taxonomic levels e.g., Genus and Species, the number of taxa recovered by every hypervariable region decreased for almost all the regions except the V3 region.

According to this finding, the analysis of bacterial diversity in the studied samples was based on concatenated sequences of hypervariable regions of the 16S rRNA.

### Bacterial diversity analysis using concatenated sequences of hypervariable regions in the studied samples

**Figure 3:**
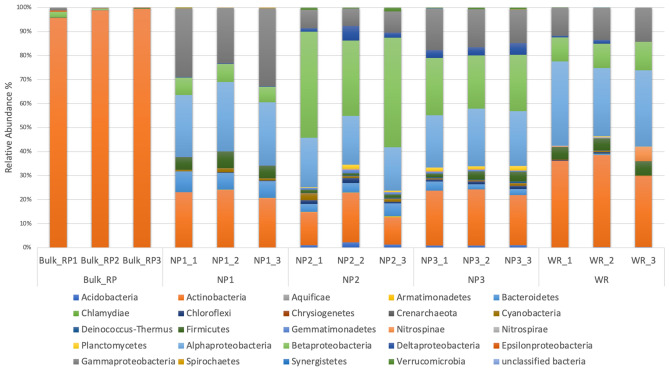
Relative abundances of Bacterial composition at phylum level in Bulk rock phosphate samples (Bulk_RP) and rhizosphere samples of phosphate native plants Alternanthera caracasana (NP1), Digitaria sanguinalis (NP2), Dittrichia Viscosa (NP3) and wheat crop (WR).

16S amplicon sequencing analysis of the studied samples included bulk phosphate mine, rhizosphere of three native wild plants in phosphate mine and wheat rhizosphere. Figure 3 presented the distribution and abundance of bacterial phyla in the studied samples using all variable regions of the 16s rRNA gene. Detailed distribution of bacterial taxa at Family and genus levels is presented in Fig. S1 available in the supplementary material. Results of the bacterial composition clearly showed differences according to sample types and the plant species. Bacterial composition of the samples obtained from bulk phosphate mine in the vicinity of the wild plants was dominated by *Actinobacteria* with average relative abundance of 97.73% (Fig. 3). *Proteobacteria* was Less abundant in these samples compared to *Actinobacteria.* At the family and genus levels, an average of 94.54% and 94.29% of OTUs were assigned to *Nocardioidaceae* and *Nocardioides,* respectively in these samples. This result indicated the selective presence and dominance of *Nocardioides* members in this particular environment among the bacterial diversity. As showed in Fig. 3, the bacterial composition was completely different in the rhizosphere samples of the three native plants from natural rock phosphate (NP). *Proteobacteria* phylum was almost enriched than other phyla with relative abundance of 62.24%, 71.15% and 65.61%, in the rhizosphere *Alternanthera caracasana* (NP1)*; Digitaria sanguinalis* (NP2) *and Dittrichia Viscosa* (NP3), respectively. Within this phylum, main representative classes were *Betaproteobacteria* (7.1%; 40.38%; 23.15% in NP1, NP2 and NP3, respectively), *Alphaproteobacteria* (26.84%; 19.65%; 22.85% in NP1, NP2 and NP3, respectively) and *Gammaproteobacteria* (28.05%; 7.85%; 15.68% in NP1, NP2 and NP3, respectively). *Deltaproteobacteria* were less abundant. High relative abundances of *Actinobacteria* (22.53%, 15.24%, 22.30% in NP1, NP2 and NP3, respectively) were also found in these native plant samples, however, at lower level, *Actinobacteria* were mainly represented by *Actinomycetales* order (21.81%; 12.58; 19.17% in NP1, NP2 and NP3, respectively) with wide taxonomic diversity at family and genus level. It is noteworthy that the relative abundance of *Nocardioidaceae* family was significantly reduced in these samples in comparison with the bulk phosphate mine samples (Supplementary materials Fig. S1). Sequences assigned to *Actinobacteria* were further analyzed in this study. Other major phyla that were found in these samples were *Bacteroidetes* (7.57%; 4.23%; 7.63% in NP1, NP2 and NP3, respectively), and *Firmicutes* (5.82%; 1.17%; 2.83% in NP1, NP2 and NP3, respectively). Broad taxonomic diversity of minor phyla was also found in the native plants samples and included *Acidobacteria*, *Armatimonadetes*, *Chloroflexi*, *Cyanobacteria*, *Gemmatimonadetes* and *Verrucomicrobia* (Fig. 3). The wheat rhizosphere samples showed a relatively similar pattern as the bacterial composition of the rhizosphere of native plants. In these samples, the assignment of taxonomic affiliation revealed high relative abundances of *Proteobacteria* (57.42%), *Actinobacteria* (35.24%), *Firmicutes* (5.52%) and *Nitrospirae* (2.32%). Broad taxonomic diversity of minor phyla was also found in the wheat rhizosphere samples. *Actinobacteria* were mainly represented by *Actinomycetales* and *Gaiellales* orders with 15.53% and 9.36%, respectively.

**Figure 4:**
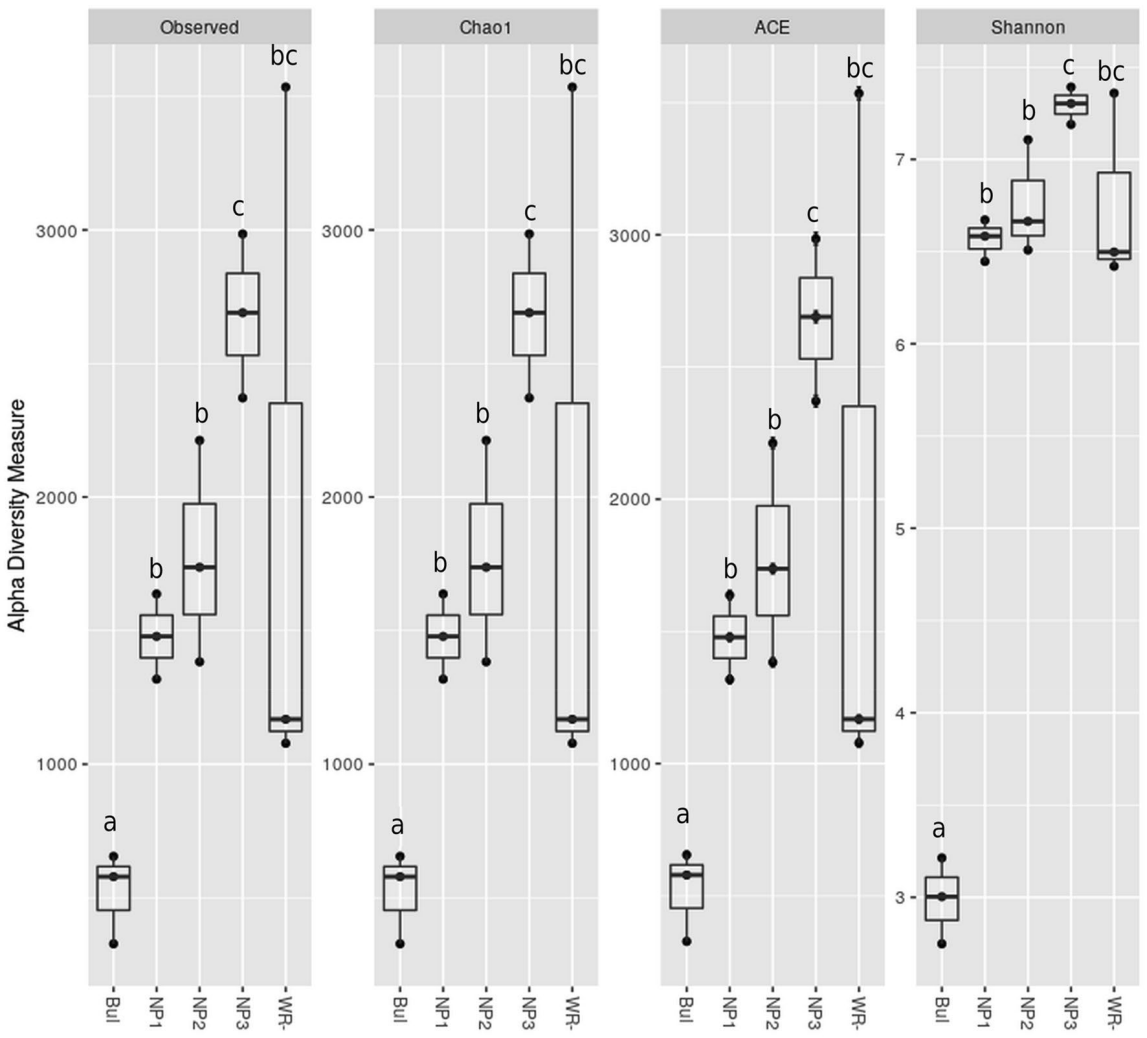
Box plot representation of Alpha diversity measures (Observed OTUs, Chao, ACE, Shannon) of bacterial communities in Bulk phosphate mines (Bul), native wild plants of the phosphate mine (NP1, NP2 and NP3) and the wheat Rhizosphere (WR) samples (n = 3 for all samples). Different letters represent significant statistical values at (P ≤ 0.05) using ANOVA test.

Alpha diversity measures (Observed OTUs, Chao, Abundance-based Coverage Estimator (ACE) and Shannon) and rarefaction curves are presented in Fig. 4 and supplementary material Fig. S2. Rarefaction analysis enabled us to gauge the extent to which total diversity had been recovered at each sample and indicated that the extent of bacterial diversity was captured by the sequencing approach. Figure 4 showed significantly lower diversity and OTUs number in the bulk mine samples. This finding was expected, because of the extreme conditions met in such habitat. Interestingly, the native plants in the phosphate mine showed higher observed OTUs, species richness and species diversity and evenness in comparison with the wheat crop rhizosphere samples, even if the observed differences were statistically not significant (Fig. 4). Higher alpha diversity measures were recorded for the rhizosphere samples of the phosphate mine native plant *Dittrichia Viscosa* (NP3), this increase was statistically significant with other native plants NP1 and NP2 samples (Fig. 4). In contrast to other samples, the wheat rhizosphere samples were characterized by variable intra-diversity, which makes the box-plot range larger than in other samples. Some replicates were highly diverse and rich in terms of species, while others were characterized by their lower richness in bacterial communities (Fig. 4).

**Figure 5:**
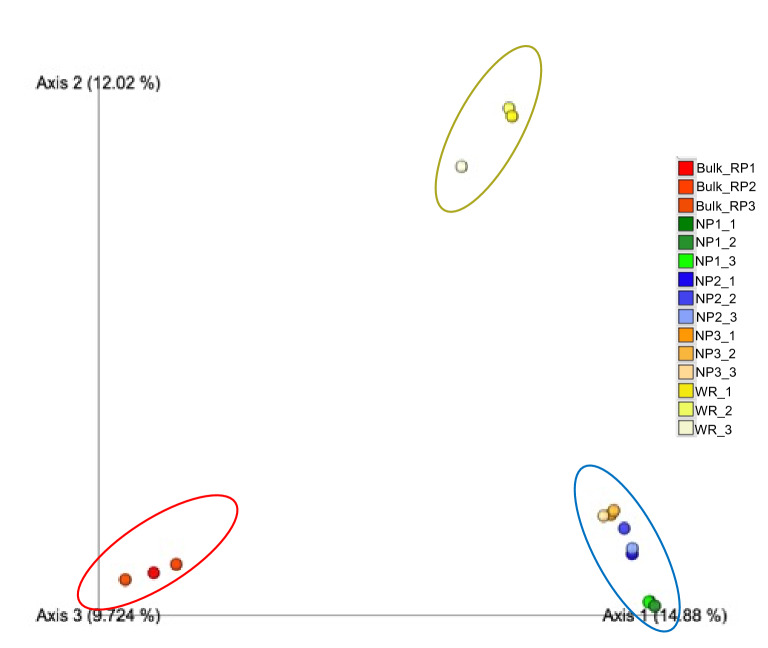
Principal coordinate analysis (PCoA) of bacterial communities from Bulk phosphate mine samples (from Bulk_RP1 to Bulk_RP3), wild phosphate mine plants Alternanthera caracasana (from NP1_1 to NP1_3), Digitaria sanguinalis (from NP2_1 to NP2_3), Dittrichia Viscosa fromNP3_1 to NP3_3) and wheat crop (fromWR_1 to WR_3). Analyses were done by using Jaccard distance matrix and visualized with emperor.

Beta diversity measures included Bray Curtis distance unweighted and weighted UniFrac distances and jaccard dissimilatory. Principal Coordinates Analysis PCoA of the latter was presented in Fig. 5 and clearly indicated differences in bacterial diversity among at least three kind of samples: the bulk phosphate mine, the rhizosphere of the native wild plants of the phosphate mine and the rhizosphere of the wheat crop. Statistical analyses using pairwise ADONIS PERMANOVA revealed significant differences between the three groups of samples.

**Figure 6:**
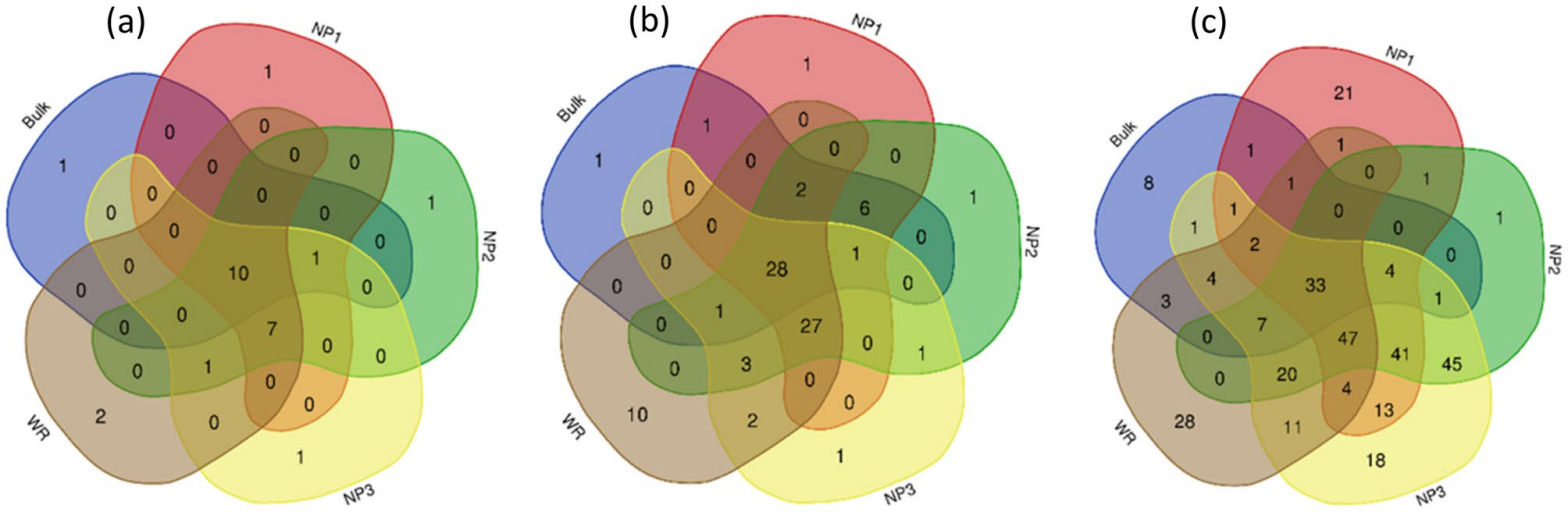
Shared OTUs at the phylum (**a**), family (**b**) and genus (**c**) level among bacterial communities in Bulk phosphate mine samples (Bulk), rhizosphere samples from wild phosphate mine plants Alternanthera caracasana (NP1), Digitaria sanguinalis (NP2), Dittrichia Viscosa (NP3) and wheat crop (WR).

The distribution of shared and unique OTUs among bacterial communities in the studied samples was presented in Fig. 6. High number of OTUs was shared between the samples at the phylum level such as *Actinobacteria, Proteobacteria, Firmicutes* and *Bacteroidetes* (Fig. 6a). The shared OTUs between the rhizosphere samples (native plants and wheat crops) included *Armatimonadetes*, *Planctomycetes* and *Verrucomicrobia,* whereas only *Nitrospirae* phylum was shared between the bulk mine samples and the rhizosphere of native plants*.* Similarly, at lower taxonomic levels e.g., family (Fig. 6b) and genus (Fig. 6c), high number of OTUs was shared between the samples (28 OTUs among 86 and 33 among 317 at the family and genus levels, respectively). Interestingly, high number of OTUs at the family level (27) were shared between the rhizosphere samples of native plants and wheat crops which included some plant-associated bacteria such as *Rhizobiaceae* and *Phyllobacteriaceae,* and indicated the effect of the plant rhizosphere in the interactions with bacterial diversity. The same statement is true for the shared OTUs at the genus level with high number among the plant samples (Fig. 6c).

### Actinobacteria phylogenetic analysis

**Figure 7:**
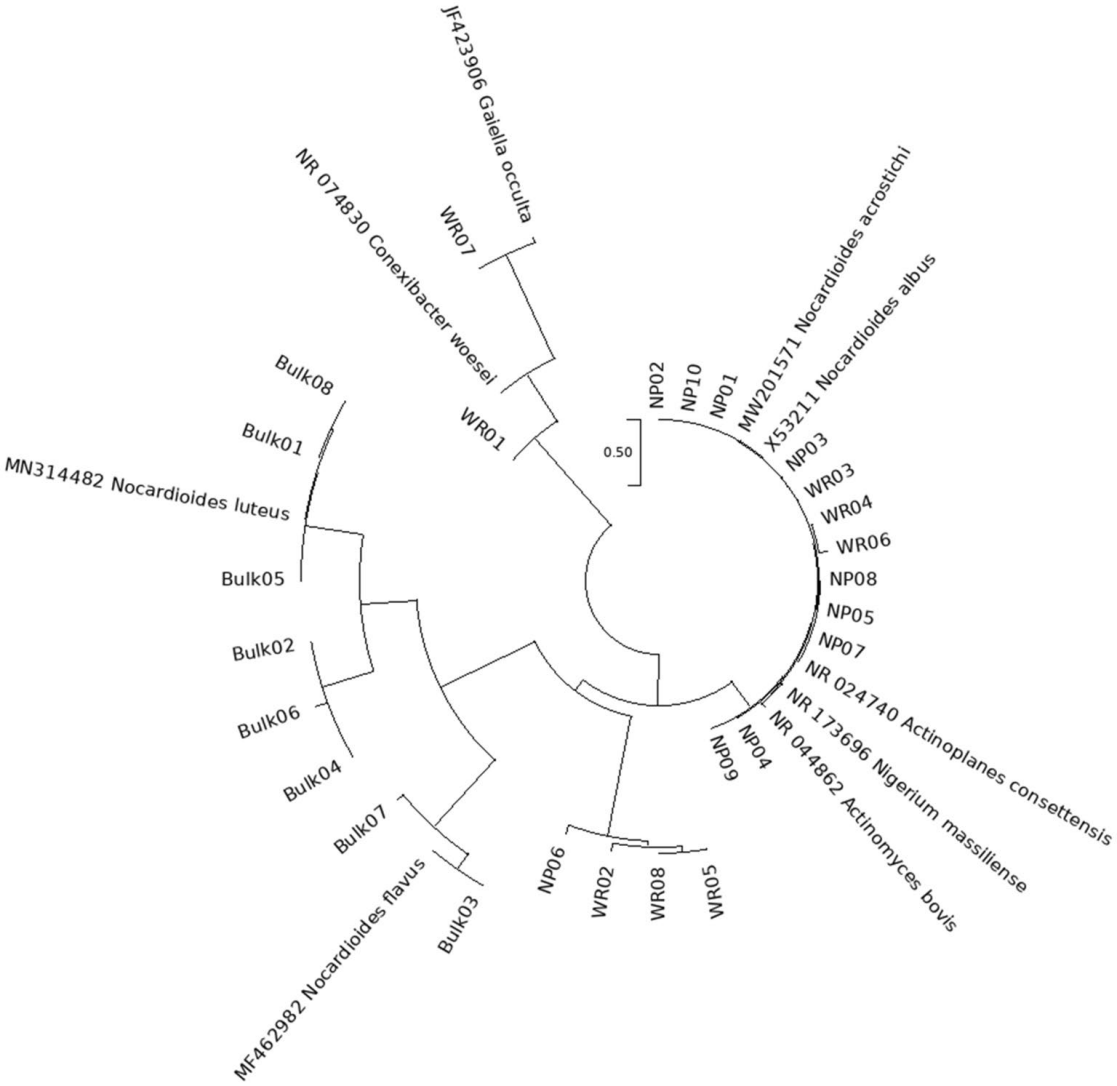
Phylogenetic tree of Actinobacteria sequences in native plants (NP01–NP09) samples, bulk phosphate mines samples (Bulk 01–Bulk 08) and wheat rhizosphere samples (WR01–WR08). The tree was generated by multiple pairwise alignment of sequences assigned to *Actinobacteria* derived from Qiime2 output using MEGAX and MAFFT Software. The phylogenetic tree was constructed by the Maximum likelihood program and a bootstrap analysis was performed with 1000 trials. Reference *Actinobacteria* species were added to the tree: *Actinomyces bovis* NR044862, *Nigerium massiliense* NR173696, *Actinoplanes consettensis* NR024740, *Nocardioides flavus* MF462982, *Nocardioides luteus* MN314482, *Nocardioides albus* X53211, *Nocardioides acrostichi* MW201571, *Gaiella occulta* JF423906 and *Conexibacter woesei* NR074830.

According to taxonomy and representative sequences tables of Qiime2, the number of sequences assigned to *Actinobacteria* varied between samples. Bulk phosphate mine samples accounted for 168 sequences assigned to *Actinobacteria*, whereas 336 sequences were assigned to *Actinobacteria* in the rhizosphere samples of the wild plants in phosphate mine. The phylogenetic tree obtained from those entire sequences is presented in supplementary materials Fig. S4. Figure 7 showed the phylogenetic relatedness and the inferred evolutionary relationships between selected *Actinobacteria* sequences in the studied samples and reference species, and revealed that *Actinobacteria* in Bulk RP were phylogenetically different and separated from those in native plant samples. Interestingly, *Actinobacteria* clades didn’t overlap between these samples and *Actinobacteria* related sequences in the bulk phosphate mine samples yielded three different clades whereas, those in native plants samples were more diverse with many branches (supplementary materials Fig. S4). *Actinobacteria* related Sequences in NP1 and NP3 were the most closely related among all samples with a common node of 72%. *Actinobacteria* in the Bulk phosphate samples were less evolutive than the sequences in the native plants samples which could be maybe related to adaptation and co-evolution process between the bacteria and the plants. All together these results indicated the selection of the *Actinobacteria* by plants in their rhizosphere and the absence of possible migration of some *Actinobacteria* from the bulk mine samples to the plants rhizosphere in their surrounding environment.

### Prediction of bacterial functions enriched in the samples

**Figure 8:**
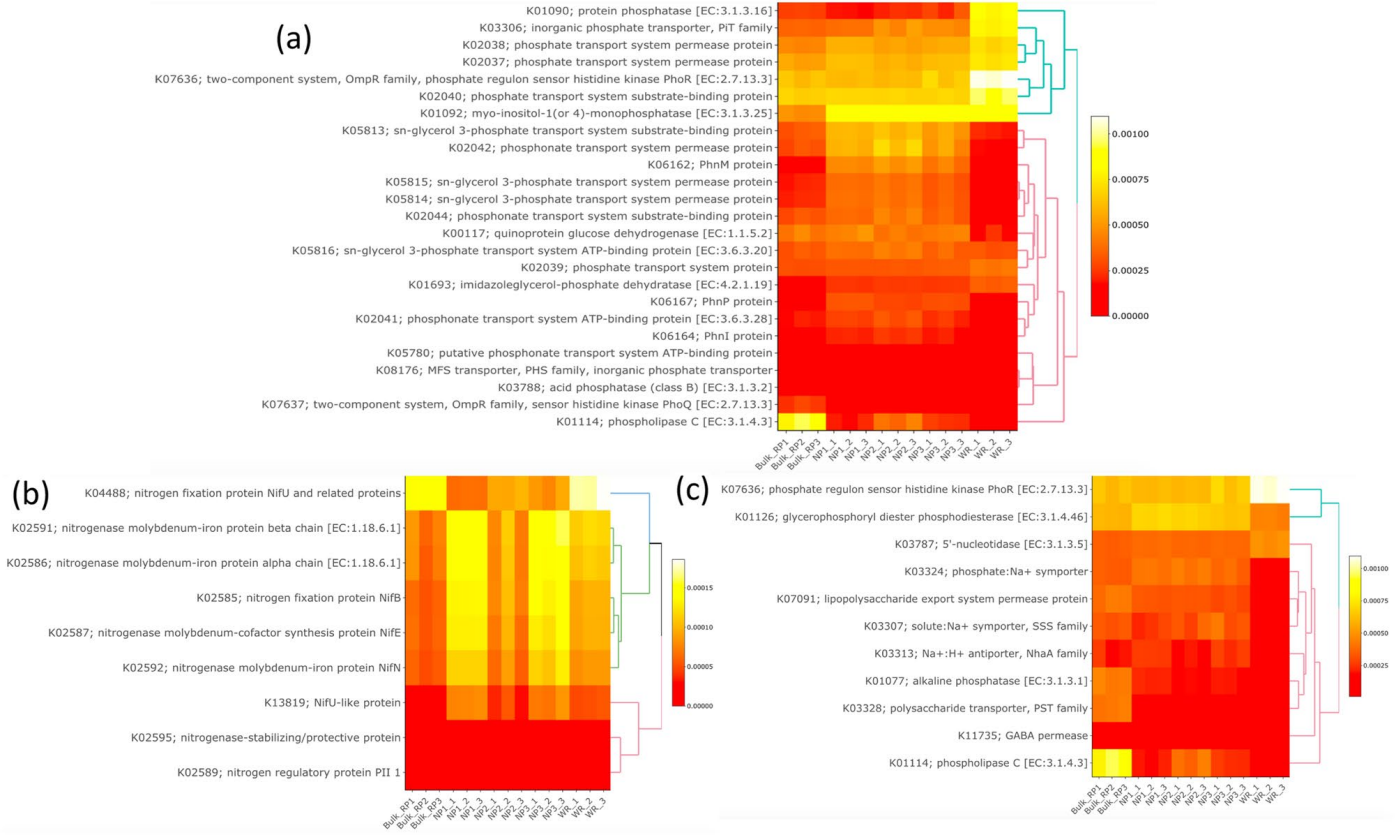
Heatmap representations of predicted bacterial functions involved in phosphate metabolism (**a**), nitrogen metabolism (**b**) and stress (**c**) across the samples Bulk phosphate mine (from Bulk_RP 1 to 3), Native plants (NP1, NP2 and NP3) and the wheat plant Rhizosphere (from WR 1 to 3) KEGG identifiers derived from KGG database developed by Kanehisa and Goto^66^.

The Bioinformatic tool Tax4fun was used in this study to predict bacterial functions involved in nitrogen and phosphorus metabolism and stress across the studied samples (Fig. 8). Figure 8b showed the enrichment of some nitrogen fixation functions, mainly in the bacteria of the rhizosphere of the phosphate mine native plants *Alternanthera caracasana* (NP1) and *Dittrichia Viscosa* (NP3). The enriched bacterial functions in these samples included nitrogen fixation proteins (mainly NifQ, NifB, NifN, NifT, NifX, NifZ, NifE) and nitrogenase cofactors. The nitrogen fixation protein NifU was mainly enriched in bacteria assessed in wheat rhizosphere and the phosphate mine native plant *Digitaria sanguinalis* (NP2). Nitrogen regulatory proteins PII 1 and PII 2 KEGG were also enriched in bulk phosphate samples and some native plants and are linked to the regulation of carbon metabolism under conditions of low nitrogen availability. Heatmap of the bacterial functions involved in phosphate metabolism (Fig. 8a) showed the enrichment of 11 predicted KEGG functions involved in mineralization, solubilization and transport (Phosphatase, acid phosphatases and inorganic phosphate transporter) among bacterial communities in the bulk phosphate mine samples. Interestingly, the assigned bacteria to the rhizosphere of native wild plants showed similar patterns, with the enrichment of many functions involved in the mineralization, solubilization and transport of phosphate. In the wheat rhizosphere samples, phosphate transport functions were mainly enriched with some phosphatase involved the phosphate mineralization processes. Figure 8c showed the enrichment profiles of bacterial functions related to stresses, it indicated that the Na^+^/H^+^ antiporters NhaC and NhaB, GABA permease, anion: H^+^ symporter DASS family, 1-aminocyclopropane-1-acyteltransferase, lipopolysaccharide O-acyteltransferase, polysaccharides transporter PST family, lipopolysaccharides export system permease protein, lipopolysaccharides biosynthesis protein WzzE, acyl homoserine lactone synthase and acyl homoserine lactone efflux proteins, were highly enriched in phosphate rock samples compared to rhizospheric soil of native plants in phosphate mine and the rhizospheric soil of wheat crop. On the other hand, all of these bacterial functions are higher in the rhizospheric soil of native plants compared to the rhizospheric soil of wheat crops. On the other hand, the NhaC family and phosphate:Na^+^ symporter were enriched in the rhizospheric soil of native plants than in bulk phosphate samples.

## Discussion

In this work we adopted a cost effective approach to decipher the bacterial communities distributions and functions in soil samples. We used the Ion 16S™ kit to assess the six hypervariable regions of the 16S rRNA (V2, V3, V4, V6-7, V8 and V9) simultaneously, in the taxonomic assignment, abundance and distribution of the bacterial communities in the studied samples. The prediction of the bacterial functionalities based on 16S rRNA profiles was performed using Tax4fun software package to highlight any possible interesting bacterial functionalities that open perspectives for in depth future soil microbiome studies in phosphate mine.

With the advancement, the accessibility and the affordability of sequencing technologies, the use of high throughput sequencing platforms in soil microbial ecology has become popular and revolutionized our understanding of the soil–plant–microorganisms interactions. However, Nannipieri et al.^31^ discussed and criticized the use of DNA-based tools in the determination of soil microbial diversity and functionalities. Schöler et al.^32^ highlighted in their opinion paper biases and pitfalls in the steps of generation, processing and interpretation of 16S rRNA sequencing data in the field of soil and plant microbiome studies. They clearly recommended the sequencing of the entire 16S rRNA gene using some new long read sequencing platforms in comparison to shorter amplicons which are mostly generated by Illumina sequencing.

To our knowledge, the present work is the first study dealing with soil bacterial DNA that used simultaneously processed multiple hypervariable regions of the 16S rRNA gene. Barb et al.^21^ used multiple hypervariable regions of the 16S rRNA gene to study the human microbiome and concluded that the use of multiple regions improve the results of identifying bacteria that compose complex communities such as those inhabiting human. The Ion 16S™ kit was also used to evaluate the performance of individual hypervariable regions of the 16S rRNA gene, for capturing human vaginal microbiota^33^. Authors of this study indicated that analyses using the V3 region generally indicated the highest bacterial diversity followed by the V6–V7 and V4 regions, while the V9 region gave the lowest bacterial resolution. With a similar way, we used in our study the Ion 16S™ Kit with both genomic DNA from ZymoBIOMICS mock standard sample and DNA extracted from soil samples originated from different habitat mainly the Moroccan phosphate mine and native plants in the ore. The obtained results in the mock samples presented in Fig. 1 were in accordance with the findings of Barb et al.^21^ and Sirichoat et al.^33^, since the hypervariable regions V2, V3, V4 and V6-7 regions gave the best taxonomic profiling up to both family and genus levels. V9 region gave less resolution and identified only *Proteobacteria* phylum. However, the percentage of taxa identified by each hypervariable region of the 16S rRNA in the DNA of the studied samples is variable according to the taxonomic level and the sample origin. In this case, V3, V4 and V67 performed better in the taxa identification at different levels. On the other hand, V9 and V8 gave the lesser profiles (Fig. 2). This finding could also corroborate the suggestion of Barb et al.^21^ which stated that the Ion 16S™ Kit does not perform well on the end of the 16S rRNA gene. Fuks et al.^34^ proposed a method called Short MUltiple Regions Framework (SMURF), to combine sequencing results from different PCR-amplified regions to provide one coherent profiling in order to improve the resolution of bacterial profiling. Recent study benchmarked a new 16S full-length-based synthetic long-read (sFL16S) technology that enables long-read sequencing by using an existing Illumina short-read sequencer and the general 16S rRNA amplicon (V3–V4) sequencing methods^35^. These studies, clearly indicated and recommended the necessity to consider the entire hypervariable regions rather than partial analysis of the 16S rRNA gene in the characterization of the diversity and composition of the microbial communities inhabiting specimen.

In our study, we characterized the bacterial communities structure in the phosphate ore (Bulk_RP) and we compare this bacterial diversity with the rhizosphere samples of some spontaneous wild plants within the phosphate ore (*Alternanthera caracasana* (NP1)*; Digitaria sanguinalis* (NP2) *and Dittrichia Viscosa* (NP3)) along with the rhizosphere of wheat in cropping system (WR). For this purpose, we used the concatenated sequences of six hypervariable regions of the 16S rRNA gene. Interesting findings are provided in this work. Indeed, the *Actinobacteria* accounted for more than 97% in the ore bulk samples with significantly lower bacterial diversity expressed by the alpha diversity indices (Fig. 4), and the enrichment of particular family and genus e.g., *Nocardioides. Actinobacteria* are generally ubiquitous and have the ability to survive under extreme conditions. They form mycelia and/or produce spores to reach water and nutrients and to survive in these environments. They can also influence the weathering of mineral elements^36^. Many reports describing the bacterial diversity in extreme habitats e.g., Chilean Deserts, Arabian peninsula desert, alpine pants and contaminated mine tailings are in accordance with our study and found *Actinobacteria* among the dominant phyla in these habitats^23,37–39^. Members of *Nocardioides* genus have been also isolated using cultivation-based approach from some extreme niches in Morocco and the obtained isolates showed plant growth-promoting activities e.g., phosphorus solubilization, potassium solubilization, siderophores and indole-3-acetic acid production^40^. However, Nafis et al.^40^ specified that *Nocardioides* genus was rare among the other *Actinobacteria* genera present in these extreme niches which needs to be evaluated in light of our results showing that this genus was highly abundant.

A shift in the bacterial communities composition was observed between the bulk rock phosphate mine samples and the rhizosphere of native plants grown in this niche (Fig. 3, supplementary material Fig. S1). This finding confirmed the roles of root exudates in recruiting and shaping the plant rhizosphere microbiome which is intensively reported^41,42^. This shift was translated by the enrichment of *Betaproteobacteria, Alphaproteobacteria, Gammaproteobacteria, Bacteroidetes* and *Firmicutes* with the appearance of new phyla mainly *Verrucomicrobia *(Supplementary materials Fig. S3). *Actinobacteria* were also found with high relative abundance in the native plants rhizosphere however the *Nocardioides* genus was lower than newly enriched taxa within *Actinomycetales* and *Gaiellales* orders (supplementary material Fig. S1). Alpha diversity measures presented in Fig. 4 indicated low abundance of observed OTUs and diversity in the bulk samples compared to the rhizosphere samples of the natives plants in the phosphate mine. Generally, studies indicated that the bulk soil under “normal conditions” is more abundant and diverse in OTUs than the rhizosphere part and plants recruit best adapted taxa^43–45^. However, Zhang et al.^46^ reported similar results in the study of rhizosphere microbial communities diversity of seagrasses *Thalassia hemprichii* and *Enhalus acoroides* in the Xincun Bay in China. One explanation provided by the authors is that bulk sediments are characterized by lower organic matter contents compared to seagrass beds. Lower organic matter and lower humidity are also the case of phosphate mine ore in the studied area compared to the rhizoplane where plants create favorable microenvironment for higher bacterial diversity. Many factors are involved in the plant recruitment of microbial communities such as soil type, edaphic conditions and the host plant genotype^47^. Results of beta diversity presented in Fig. 5 indicated significantly separated bacterial diversity between the native plants, the bulk mine and the wheat crop with insignificant differences between the three studied native plants. This result may suggest the influence of the soil conditions rather than the genotype of the host plant in the shaping of bacterial communities in this particular environment. Such suggestion is in accordance with the conclusion of Eida et al.^39^ who showed that the host desert plants phylogeny aligns to a lesser degree with the rhizosphere bacterial communities which are largely determined by the soil conditions. In this particular habitat, selective pressures are exerted to plants with adapted genotype and their associated bacterial communities to choose the best adapted phyla to help them thrive in such conditions^45^. Furthermore, it was intrigant to investigate the occurrence of some OTUs in the rhizosphere of native plants through the phylogenetic analysis of *Actinobacteria* related sequences. We found interesting separation of clades according to the sample origins (bulk phosphate vs native plants rhizosphere) (Fig. 7) which opens hypothesis to explain the origin of *Actinobacteria* diversity in the studied samples mainly in the rhizosphere of the ore native plants. We reported high relative abundance of *Nocardioides* genus in the bulk samples exceeding 90% and which drastically decreased in the rhizosphere samples of the native plants, where we found more diversity within the *Actinomycetales* order. This diversity could be attributed to a plants-driven factors that determine the dominant bacterial populations in their rhizosphere^48^. Another explanation could be the occurrence of distinct ecotypes in the wild plants rhizosphere that have co-evolved. In fact, Lopes et al.^49,50^ showed in two different specific studies distinct populations of *Pseudomonas koreensis* and *Pseudomonas putida* between sugarcane rhizosphere or bulk soil and concluded that the divergence of these populations might have resulted from the differential selective pressures posed by the bulk soil versus rhizosphere habitats even in the populations of same species. Furthermore, the effect of microbial seed load on rhizosphere and endosphere bacterial communities was investigated in wheat by Kavamura et al.^51^. In fact, plant seeds harbor diverse microbial communities and their composition is determined by plant genotype, environment, and management practices^52^. The seed borne microbiome transmission is still poorly investigated and links between seed and soil microbiomes are not fully understood. Such transmission could also explain the diversity of bacterial communities in the rhizosphere of the native plants studied in the phosphate ore. In any case, further specific studies are needed to investigate the occurrence of bacterial diversity in different part of plants and the surrounding environment mainly in extreme contexts.

In this study, we predicted the functional profiles of bacterial taxa based on 16S rRNA sequencing data using the Tax4Fun bioinformatic tool (Fig. 8). This approach could be adapted in low cost studies based on the 16S amplicon sequencing rather than the expensive shotgun metagenomics. It could also give insights into some particular bacterial functions under different context and conditions that could be targeted in subsequent studies. The obtained results, presented in Fig. 8, showed some interesting findings. For example, the enrichment of the nitrogenase functions and the nitrogenase molybdenum-iron (MoFe protein) in the rhizosphere of native plants in the nitrogen-deprived phosphate mines, explained the recruitment by these plants of atmospheric nitrogen-fixing bacteria to ensure its nitrogen requirements. Such functions were not found in rock phosphate bulk samples without plants, which strengthened the recruitment hypothesis. In wheat plants rhizosphere, these functions were less enriched since this environment is rich in nitrogen coming from fertilizers. The nitrogen fixation process is based on the transfer of electrons from the Fe protein to FeMo-co, in which a nitrogen molecule is converted to two ammonia molecules. The biosynthetic process of all proteins involved in this mechanism such as FeMo-co are extremely vulnerable to environmental and endogenous oxygen. The aerobic and micro-aerobic nitrogen-fixing organisms need to create strict anaerobic microenvironments in the cell to facilitate active nitrogenase functions. To meet this need, special accessory enzymes / proteins are required for the biosynthesis of clusters and mainly FeMo-co such as the genes products of nifH, nifQ, nifB, nifE, and nifN and nitrogen fixation proteins NifX involved in efficient transfer processes of NifB-co^53,54^. In this sense, NifZ is involved in the maturation of the P-cluster, the second cluster of catalyzing the biological nitrogen fixation reaction by nitrogenase. On the other hand, the expression of the nitrogen regulatory proteins PII 1 and PII 2 are linked to the regulation of carbon metabolism under conditions of low nitrogen availability. In bacteria, PII proteins control target proteins in response to cellular ATP/ADP levels and the 2‐oxoglutarate status, thereby coordinating the cellular carbon/nitrogen balance. In addition to their involvement in the regulation of carbon metabolism, these proteins are able to control the activity of acetyl-CoA carboxylase and glutamine synthetase (GS) in response to the availability of nitrogen sources^55^. The resulted prediction functions explain the hypothesis of recruitment of nitrogen-fixing bacteria by native plants under nitrogen starvation, while ensuring the control of carbon metabolism and energy for proper functioning of nitrogenase under these stress conditions. Similarly, prediction of bacterial functions related to the solubilization and assimilation of phosphate revealed the enrichment of specific functions according to samples nature. In rock phosphate bulk samples, several functionalities were enriched such as low-affinity inorganic phosphate transporters, acid phosphatase, 3-phytase, glucose-1-phosphatase, alkaline phosphatase, phospholipase, phosphonoacetaldehyde hydrolase, as well as phosphate regulon PhoB (Fig. 8a). Enrichment of these functions could be explained by the low availability of inorganic phosphate in the rock phosphate, and the bacterial populations showed these adaptation mechanisms under this harsh environment such as the activation of low-affinity inorganic phosphate transporters. At the same time, the activation of the solubilization potential of phosphate via the expression of the gene products of phosphatases such as acid phosphatase, 3-phytase, glucose-1-phosphatase, alkaline phosphatase, phospholipase, phosphonoacetaldehyde hydrolase. However, due to the dephosphorylating action, acid phosphatase activity could indirectly influence inorganic P solubilization by lowering the medium pH^56^. In the same way, phosphate regulon PhoB which is induced by phosphate deprivation was also enriched in the phosphate rock samples. The Phosphate (Pho) regulon is a bacterial regulatory mechanism used for the conservation and management of inorganic phosphate within the cell^57^. In the other hand, Bacterial high affinity transport systems are involved in active transport of solutes across the cytoplasmic membrane. Most of the bacterial ATP-binding cassette importers are composed of one or two transmembrane permease proteins, one or two nucleotide-binding proteins and a highly specific periplasmic solute-binding protein^58^. This explained the prediction results of the enrichment of functions involved in activation of active transport in the case of wheat crops, such as the phosphate transporters, as well as the enrichment of The inorganic phosphate transporters (PiT) family which appear to catalyze inorganic phosphate (Pi).

Results of the stress related bacterial functions enrichment showed that they were higher in the bulk phosphate mine samples and in the rhizosphere of native plants compared to wheat crop. Looking at the role of these stress related functions presented in Fig. 8c, we find that the expression of these functional genes is linked to stress tolerance. Na^+^ and H^+^ are the most common ions and they play primary roles in cell physiology: both are important in cell bioenergetics. Indeed, when the concentration of these ions becomes too high or too low they turn into potent stressors to cells. every cell has a very efficient homeostatic mechanism for these ions. Proteins that play a primary role in this homeostatic mechanism are the Na^+^/H^+^ antiporters. NhaC, the key Na^+^/H^+^ antiporter, plays a principal role for regulation of alkaline phosphatase biosynthesis, which has the role of solubilization of phosphate in alkaline conditions of rock phosphate. In the case of the rhizospheric soil of native plants existing in the phosphate mine, the low enrichment of this function could be explained by the change in soil conditions thanks to the root exudates rich in organic acid, amino acids and others metabolites and organic carbon. This explains the increase in microbial diversity in rhizospheric soil of these native plants compared to that of phosphate mine presented in alpha diversity measures. Low-molecular-weight organic compounds in root exudates play a key role in plant–microorganism interactions by influencing the structure and function of soil microbial communities. Under these conditions of nitrogen and carbon stress in the phosphate rock, the GABA permease function was highly enriched. This enzyme is responsible for the release of GABA which plays an important role in the regulation of nitrogen uptake, and at the same time considered as carbon source under stress conditions^59^. On the other hand, bacterial surface molecules known as lipopolysaccharide (LPS), produced by most Gram-negative bacteria, create a permeability barrier at the cell surface and is a main contributor to the innate resistance that Gram-negative bacteria display against many stresses^60^. This postulate was supported by the high enrichment of lipopolysaccharide O-acyteltransferase, polysaccharides transporter PST family, lipopolysaccharides export system permease protein, lipopolysaccharides biosynthesis protein WzzE in rock phosphate samples considered as a stressful environment. In addition, soil-dwelling bacteria collectively referred to rhizobia synthesize and perceive N-acyl-homoserine lactone (AHL) signals to regulate gene expression in a population density-dependent manner^61^. This signaling mechanism is called Bacterial quorum sensing (QS) involved in many physiological processes in bacteria^62^. According to this analysis, we can say that the functions expressed by the bacterial community in rock phosphate are linked to the tolerance and survival of these microorganisms in this extreme environment. The decrease of these functions linked to the increase in the expression of other functions in rhizospheric soil of these extremophilic native plants could be explained by the change in the conditions of this micro-environment by the root exudates rich in organic acid and other metabolites which can influence the pH, the availability of a carbon source that allows a new recruitment of other microbial populations ensuring others functions such as atmospheric nitrogen fixation, phosphate and other nutrients solubilization.

To our knowledge, the present work is the first study dealing with soil bacterial DNA that used simultaneously processed multiple hypervariable regions of the 16S rRNA gene. The proposed amplicon sequencing approach combined to bioinformatic prediction of bacterial functions could be considered as low cost method to study the bacterial communities in soils and plants. The application of such approach to samples from the phosphate mines soil and wild plants in this environment revealed the need to consider multiple hypervariable regions of the 16S rRNA gene. In the taxonomic profiling of bacterial diversity in soil and plant samples we concluded the need to consider the V3–V4 and V6–7 regions rather than the widely used V3–V4 primers and the V9 region identified only *Proteobacteria* phylum.

Application of the above mentioned approach to bulk samples from Moroccan phosphate mine and the rhizosphere of some native plants in this environment along with wheat plants in cropping system revealed interesting bacterial diversity. The distribution of different bacterial taxa at different levels was governed by the plant genotypes and to wider extent by the local conditions. The plants in the phosphate mine were able to enrich and/or recruit new phyla not present in the bulk samples. Regarding the origin of the bacterial diversity in the native plants, we hypothesized possible recruitment and co-evolution of these taxa with their hosts and/or the seed borne microbiome transmission to the plants. Such hypothesis need further investigations. Interesting predicted bacterial functionalities were also revealed using bioinformatic tools. The functions were involved in plant growth and development and stress tolerance in extreme environments. These functions were enriched and associated with the bacterial taxa distribution both in the bulk and native plants in phosphate mine samples more than the wheat rhizosphere samples. This opens a paramount opportunities to target and investigate the interesting identified phyla and the predicted bacterial functionalities in the wild plants to develop new generation of selected bacterial inoculants for sustainable agriculture.

## Methods

### Sampling and experimental design

**Figure 9:**
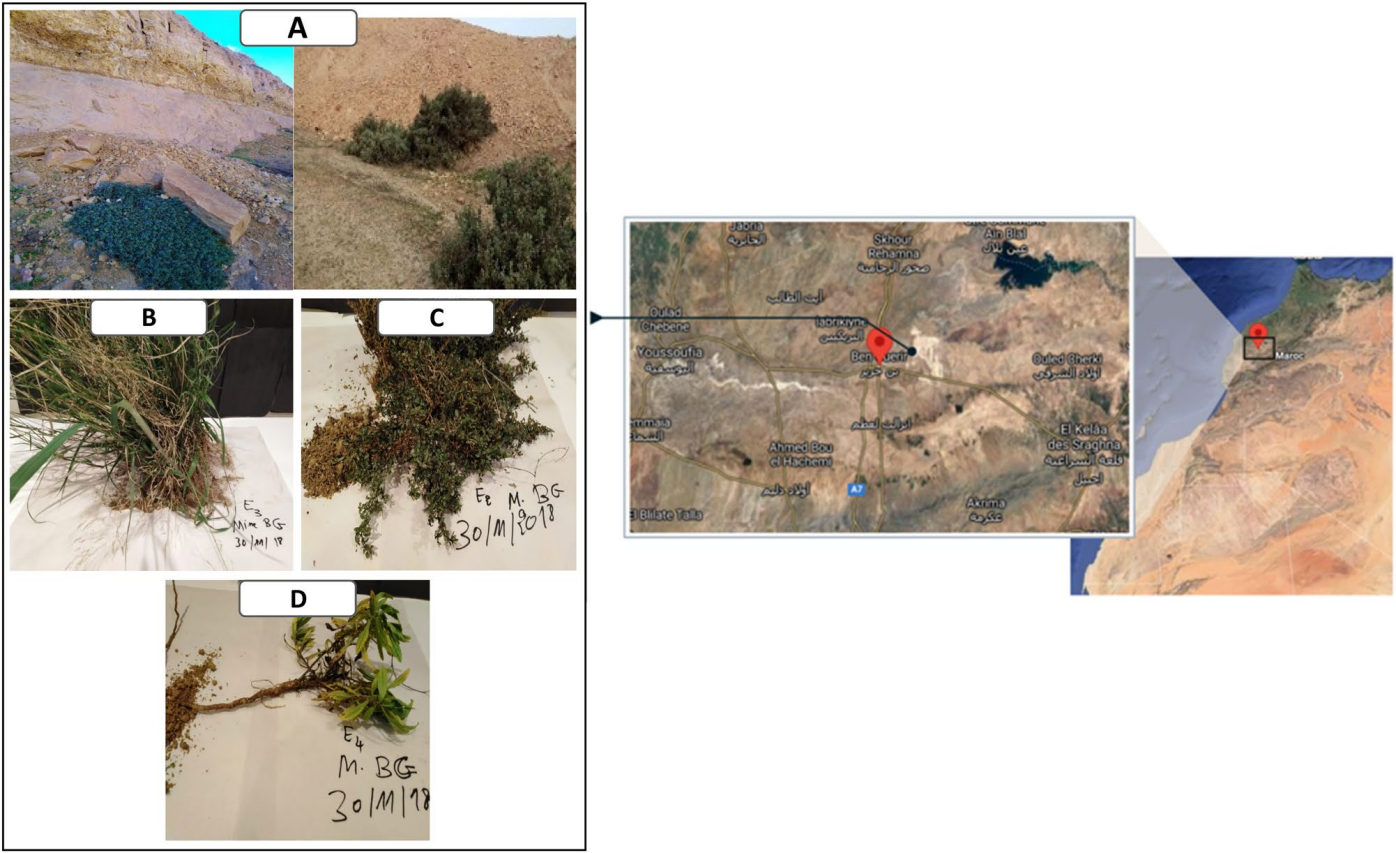
Geographic location (google map images version 2021—https://www.google.fr/maps/@32.2333612,-7.8047697,3347m/data=!3m1!1e3) and Samples of Moroccan phosphate mine (**A**) rock phosphate mining site—Ben Guerir—Morocco; (**B**) NP1: *Alternanthera caracasana*; (**C**) NP2: *Digitaria sanguinalis*; (**D**) NP3: *Dittrichia Viscosa.*

In this project, 15 soil samples were collected from the sampling sites that include the bulk soil of the phosphate ore in Ben Guerir city in Morocco (N 32°13′28.81533; W 7°50′22.412″) (Fig. 9A), soil rhizosphere of three native plants in the ore that spontaneously grow in this environment (Fig. 9B–D) and the rhizosphere of wheat plants in cropping system in the region of Sidi Allal Tazi, Kénitra city in Morocco (N 34°31′30.5394″; W 6°19′11.8164″). Three independent samples were taken from the bulk soil of the phosphate mine and named Bulk_RP1, Bulk_RP2 and Bulk_RP3. Three native plants were also collected and three independent samples were taken from the soil loosely attached to their roots as follow *Alternanthera caracasana* (Fig. 9B) NP1_1, NP1_2 and NP1_3; *Digitaria sanguinalis* (Fig. 9C) NP2_1, NP2_2 and NP2_3 and *Dittrichia Viscosa* (Fig. 9D) NP3_1, NP3_2 and NP3_3. Three independent sample were also taken from the durum wheat (*Triticum aestivum* L. ssp. *durum*) plants rhizosphere and named WR_1, WR_2 and WR_3. All samples were collected manually under aseptic conditions at a depth of 10–20 cm around the roots, placed into sterile tubes and plastic bags, transported to the laboratory at 4 °C and stored at − 20 °C until the DNA extraction. The use of plant/plant material in the study complies with relevant institutional, national, and international guidelines and legislation.

Additionally, a mock sample control consisting of the ZymoBIOMICS™ Microbial Community Standard (supplementary materials Fig. S5), which contains samples from 8 well-known species of bacteria and 2 yeasts was run concurrently along with the soil samples, to assess data quality and the taxonomic profiling of the applied sequencing strategy.

### DNA extraction and metagenomic analysis

Genomic DNA was extracted from the 15 samples using DNeasy^®^ PowerSoil^®^ Kit (Qiagen, 12888), following the manufacturer’s procedure. The purity calculated by the absorbance ratio A_260_/A_280_ and A_260_/_230_ and quantity of DNA samples were determined using NanoDrop 2000 UV–Vis spectrophotometer (Thermo scientific, USA) and Qubit™ 4 Flurometer (Thermo scientific, USA), respectively. The 16S Metagenomic sequencing library preparation protocol (Thermo-Fisher Scientific, USA) was followed for the DNA library preparation. For each sample, the six hypervariable regions of the 16S rRNA gene were amplified using two separate PCR reactions with two specific primer sets V2-4-8 and V3-6,7-9 of the kit Ion 16S Metagenomics (Thermo-Fisher Scientific, Cat. no. A26216). Each 30 µl PCR mix contained 15 µl 2X Environmental Master Mix, 3 µl 16S rRNA Primer set and 12 µl DNA sample. Cycling conditions were as follows: denaturation at 95 °C for 10 min, 25 cycles of 30 s, annealing at 56 °C for 30 s and 20 s extension at 72 °C. Negative control (water) and positive control (*E. coli* DNA) reactions were used to confirm the amplification of 16S rRNA and equal volumes of the 2 PCR reactions were combined. Final DNA library was made from 10 to 100 ng of combined amplicons using the Ion Plus Fragment Library Kit (Thermo-Fisher Scientific, Cat. no. 4471252) and Ion Xpress Barcodes Adapters, 1–16 (Thermo-Fisher Scientific, (Thermo-Fisher Scientific, Cat. no. 4471252) following recommendations of the manufacturer. Adapter-ligated and nick-repaired DNA was purified using × 1.4 volumes of Agencourt AMPure beads (Beckman Coulter, USA) and eluted in Tris–EDTA buffer. The optimal library concentration for template preparation was estimated by qPCR (Applied Biosystems^®^7500 Fast) using the Ion Universal Library Quantitation Kit (Thermo-Fisher Scientific, Cat. no. A26217). Each library was adjusted to a concentration of 10 pM and equal volumes of each library were pooled. The Ion Chef ™ instrument was used to clonally amplify the pooled library on nanosized ionosphere particles by emulsion PCR. The bead enrichment and the loading of Ion 318TM Chip Kit v2 were also conducted using The Ion Chef ™ instrument. The sequencing was run on the Ion PGM™ System using the Ion PGM™ Sequencing 400 Kit according to the manufacturer protocol.

### Basecalling and bioinformatics analysis of sequencing data

Basecalling and demultiplexing were performed with the Torrent Suite™ Software version 5.0.5 (Life Technologies) using recommended parameter settings. DNA sequencing data were collected in FASTQ format, analyzed using QIIME 2 v2020.8^62^. Briefly, Raw torrent ion sequence reads were imported from QIIME2 using the "Sample Data” (Sequences with Quality). Then denoised and filtered with the dada2 pipeline to eliminate noisy and chimeric sequences, demultiplexed, and construction of noised paired-end sequences. Taxonomic identification was performed using Curated MicroSEQ^®^ 16S Reference Library v2013.1 database based on Curated Greengenes v13.5, threshold value for percentage identity for genus and species ID was 97%.

All the basic measures used in the analysis of alpha and beta diversity were calculated on the basis of the rooted phylogenetic tree. In addition to observed OTU richness via alpha diversity, we used the phylogenetic diversity of Faith'DP (a phylogenetic measure of diversity based on total branch length of the bacterial 16S rRNA gene phylogeny captured by each sample)^63^. The sampling depth of 100 readings, a reason to normalize the variance, which excluded four samples. Among these, the Kruskal–Wallis test (in pairs, p-value 0.05) was used to assess the statistical significance of alpha diversity. For Beta diversity analysis, following QIIME2 diversity plugin, OTUs were identified with qualitative (Jaccard and unweighted UniFrac) and quantitative (Bray–Curtis and weighted UniFrac) distance measurements at a sample depth of 100 readings. The statistical significance between the different groups was assessed by the permutation-based ANOVA (PerMANOVA) test^64^ with 999 permutations (beta group significance command in the diversity plugin). Thereafter, Principal coordinate analysis (PCoA) plots were generated by the QIIME2 Emperor tool to explore the structure of the bacterial community. The bar graphs showing the taxonomy levels were generated by the Microsoft excel tool using the OTUs tables generated by Ion Reporter™ Software version 5.18. The package phyloseq was used to perform post-analysis of Alpha and Beta diversity. The Figures were generated by ggplot package in R software version 3.5.

To separate reads into different variable regions from the 16S Metagenomics Kit we followed two strategies. In the first one we used Mothur pipeline as descripted in Barb et al.^21^ and the second by Qiime2 using specific primers for each region. For the Mothur method, briefly, we aligned the full set of reads with the Mothur script align.seqs using Silva as the reference database. The aligned report file was then submitted to an R script that counts the number of reads with the same start and stop alignment position along the reference database (Table 1). The forward and reverse reads were then visualized along the translated 16S rRNA gene coordinates and reads were grouped into their corresponding variable regions based on where they aligned.

**Table 1:** Information on the selected primer pairs used for extracting the different hypervariable regions of the 16S rRNA gene for the mothur analysis pipeline described by Barb et al.^21^.

S.No	Forward primer	Sequence of the forward primer	Reverse primer	Sequence of the reverse primer	Region
1	119	AGYGGCGNACGGGTGAGTAA	338	TGCTGCCTCCCGTAGGAGT	V2
2	357	CCTACGGGAGGCAGCAG	518	ATTACCGCGGCTGCTGG	V3
3	577	AYTGGGYDTAAAGNG	785	TACNVGGGTATCTAATCC	V4
4	785	AGGATTAGATACCCT	907	CCGTCAATTCCTTTGAGTTT	V5
5	978	TCGAtGCAACGCGAAGAA	1062	ACATtTCACaACACGAGCTGACGA	V6
6	1114	GYAACGAGCGCAACCC	1220	GTAGCRCGTGTGTMGCCC	V7
7	1070	ATGGCTGTCGTCAGCT	1385	ACGGGCGGTGTGTAC	V8
8	119	AGYGGCGNACGGGTGAGTAA	518	ATTACCGCGGCTGCTGG	V23
9	357	CCTACGGGRSGCAGCAG	798	GGGGTATCTAATCCC	V34
10	357	CCTACGGGAGGCAGCAG	907	CCGTCAATTCCTTTGAGTTT	V35
11	577	AYTGGGYDTAAAGNG	907	CCGTCAATTYYTTTRAGTTT	V45
12	805	GGATTAGATACCCTGGTAGTC	1062	ACAGCCATGCAGCACCT	V56
13	985	CAACGCGAAGAACCTTACC	1220	GTAGCRCGTGTGTMGCCC	V67
14	1065	AGGTGCTGCATGGCTGT	1391	GACGGGCGGTGWGTRCA	V78

However for Qiime2 method, each region V2; V3; V4, V6-7 and V8 was analyzed separately by the same Qiime2 pipeline described above. In addition, analysis as well as the comparison between the regions were carried out in the R environment (http://www.r-project.org/). Also, the Venn diagram was generated by R package according to different taxonomic levels and the graphical output was in the form of a Venn/Euler diagram.

### Phylogenetic analysis of Actinobacteria related sequences

To analyze the Actinobacteria sequences obtained in the Meta-taxonomics output, the Actinobacteria assigned sequences were exported from Qiime2 via the "Qiime taxa filter-seqs" command and then transformed to a fasta format to perform the phylogenetic analyses. Subsequently, the sequences of plant sequences were coded by native plants (NP), Bulk phosphate (Bulk) and wheat rhizosphere (WR) via the command "sed 's/ > NP1_*/' foo.in > bar.out". Reference sequences of some representative *Actinobacteria* species were obtained from NCBI GenBank. The output of MAFFT is imported into MEGAX^65^ to construct the phylogenetic tree by the Maximum likehoold method with a boostrap of 1000 times. We also collected the matrix of phylogenetic distance and the diversity parameters calculated by MEGAX. The phylogenetic signal with K and λ were calculated by R via package phytools.

### Predicting functional profiles from 16S rRNA data using Tax4Fun

The prediction of the functional profiles of bacterial taxa based on 16S rRNA sequencing data was performed with Tax4Fun which an open-source R package using the OTUs Table containing taxonomic data according to Silva’s database^30^. This tool was used to predict functional community profiles based on 16S rRNA data to outline bacterial metabolism in the studied samples in the phosphate mines in comparison to the wheat crop. A list of 44 KEGG functions^66^ involved in Biological nitrogen fixation (9 KEGG), phosphate metabolism (25 KEGG) and stress related functions (11 KEGG) were assessed for differentially enrichment (KeGG Onthology, KO) using non parametric T-test performed on QIIME. Results were presented as heatmap using R software version 3.5, package heatmaply.

## Supplementary Information

Supplementary Information.

## Data Availability

The datasets generated during the current study are available in the NCBI repository, under the Bioproject number PRJNA729805 available through the web link https://www.ncbi.nlm.nih.gov/bioproject/PRJNA729805. KeGG Onthology tables and additional data are available upon request.

## Abbreviations

PGPB: Plant growth promoting bacteria
ACC deaminase: 1-Aminocyclopropane-1-carboxylate (ACC) deaminase
NP: Native plant
ACE: Abundance-based coverage estimator
OTUs: Operational taxonomic units
PCoA: Principal coordinates analysis
KEGG: Kyoto encyclopedia of genes and genomes
RP: Rock phosphate
