# Supplementary material for: Investigation of bacterial diversity using 16S rRNA sequencing and prediction of its functionalities in Moroccan phosphate mine ecosystem
Source: Sci Rep. 2022 Mar 8;12:3741. doi: 10.1038/s41598-022-07765-5 (PMC8904503; doi:10.1038/s41598-022-07765-5)
Supplement: Supplementary file 1 — Supplementary Information. [file 41598_2022_7765_MOESM1_ESM.docx]

**Title: Investigation of Bacterial diversity using 16S rRNA sequencing and prediction of its functionalities in Moroccan phosphate mine ecosystem.**

**Authors:** Salah Eddine Azaroual ^1*^, Yassine Kasmi ^1,2^, Abderrahim Aasfar ^1^, Hicham El Arroussi^1^ Youssef Zeroual ^3^, Youssef El Kadiri^4,5^, Abdelali Zrhidri ^4^, Elmostafa Elfahime ^6^, Abdelaziz Sefiani ^4^, Issam Meftah Kadmiri ^1,7*^.

^1^ Green Biotechnology Laboratory, Moroccan Foundation for Advanced Science, Innovation and Research (MAScIR), Rabat Design Centre, Rue Mohamed Al Jazouli, Madinate Al Irfane, 10100 Rabat, Morocco.

^2^ Mohammadia School of Engineering, Process Engineering Research Team, Mohammed V University, Rabat, Morocco

^3^ Situation Innovation Group–OCP Group, Jorf Lasfar, Morocco

^4^ Department of Medical Genetics, National Institute of Health, Rabat, Morocco

^5^ Research Team in Genomics and Molecular Epidemiology of Genetic Diseases, Genomic Center of Human Pathologies, Faculty of Medicine and Pharmacy, Mohammed V University in Rabat, Morocco

^6^ National Center for Scientific and Technical Research/Rabat (CNRST), Angle Avenue Allal El Fassi, avenue des FAR, District Hay Ryad, BP 8027, United Nations, 10102 Rabat, Morocco

^7^ AgroBioSciences, Biodiversity and Plant Sciences, Plant and Soil Microbiome Sub-program, Mohammed VI Polytechnic University, Ben Guerir, Morocco

Contact author:

Issam Meftah Kadmiri

Green Biotechnology Laboratory, Moroccan Foundation for Advanced Science, Innovation and Research (MAScIR), Rabat Design Centre, Rue Mohamed Al Jazouli, Madinate Al Irfane, 10100 Rabat, Morocco

[i.kadmiri@mascir.ma](mailto:i.kadmiri@mascir.ma)

Phone: 00212664826263

**Supplementary Materials**

**Table S1: Summary statistics 16S rRNA metagenomics analysis**

| Samples | Toral reads | Ignored reads  (Low number of copies) | Valid reads | Mapped reads |
| --- | --- | --- | --- | --- |
| BulkRP_1 | 168998 | 33509 | 136002 | 102405 |
| BulkRP_2 | 129911 | 19859 | 103518 | 83659 |
| BulkRP_3 | 256660 | 42486 | 206123 | 163571 |
| NP1_1 | 198001 | 67922 | 109331 | 41374 |
| NP1_2 | 186496 | 63325 | 102255 | 38807 |
| NP1_3 | 143940 | 49907 | 81797 | 31833 |
| NP2_1 | 104707 | 44395 | 64594 | 19989 |
| NP2_2 | 174595 | 67408 | 97830 | 29675 |
| NP2_3 | 139399 | 52701 | 78272 | 25356 |
| NP3_1 | 236850 | 90632 | 137603 | 45794 |
| NP3_2 | 223395 | 85342 | 124515 | 38155 |
| NP3_3 | 176775 | 70066 | 97681 | 26891 |
| WR_1 | 108095 | 42222 | 56210 | 13838 |
| WR_2 | 455797 | 148694 | 232664 | 82716 |
| WR_3 | 79278 | 35531 | 44273 | 8717 |
| **Toral** | **2 782 897** | **913 999** | **1 672 668** | **752 780** |

**Table S2: Summary statistics 16S rRNA metagenomics analysis according to six hypervariable regions**

| Primer name | Valid reads | Mapped reads | Forward valid reads | Reversed valid reads | Unmapped reads |
| --- | --- | --- | --- | --- | --- |
| BulkRP_1 | | | | | |
| V2 | 17988 | 13182 | 9209 | 8779 | 0 |
| V3 | 12376 | 8677 | 6701 | 5675 | 0 |
| V4 | 47257 | 36627 | 22975 | 24282 | 88 |
| V67 | 2488 | 1405 | 1114 | 1374 | 0 |
| V8 | 55566 | 42340 | 29773 | 25793 | 0 |
| V9 | 327 | 174 | 178 | 149 | 0 |
| BulkRP_2 | | | | | |
| V2 | 1685 | 1129 | 805 | 880 | 0 |
| V3 | 65123 | 55692 | 34593 | 30530 | 0 |
| V4 | 2563 | 1576 | 1295 | 1268 | 0 |
| V67 | 31307 | 23582 | 11905 | 19402 | 0 |
| V8 | 2668 | 1618 | 1404 | 1264 | 0 |
| V9 | 172 | 62 | 116 | 56 | 0 |
| BulkRP_3 | | | | | |
| V2 | 17730 | 13140 | 9341 | 8389 | 0 |
| V3 | 69175 | 59518 | 37512 | 31663 | 0 |
| V4 | 41995 | 32638 | 20211 | 21784 | 68 |
| V67 | 29830 | 22316 | 13304 | 16526 | 0 |
| V8 | 47231 | 35918 | 24003 | 23228 | 0 |
| V9 | 162 | 41 | 84 | 78 | 0 |
| NP1_1 | | | | | |
| V2 | 4675 | 524 | 2604 | 2071 | 0 |
| V3 | 27793 | 13182 | 14967 | 12826 | 35 |
| V4 | 10767 | 2808 | 5543 | 5224 | 0 |
| V67 | 25435 | 7140 | 12777 | 12658 | 0 |
| V8 | 27594 | 8778 | 13502 | 14092 | 0 |
| V9 | 13067 | 8942 | 7835 | 5232 | 0 |
| NP1_2 | | | | | |
| V2 | 2745 | 245 | 1358 | 1387 | 0 |
| V3 | 33175 | 15880 | 17418 | 15757 | 123 |
| V4 | 8614 | 1927 | 4268 | 4346 | 0 |
| V67 | 20401 | 4252 | 9329 | 11072 | 0 |
| V8 | 25179 | 8414 | 10801 | 14378 | 0 |
| V9 | 12141 | 8089 | 8930 | 3211 | 0 |
| NP1_3 | | | | | |
| V2 | 3359 | 622 | 1733 | 1626 | 12 |
| V3 | 22680 | 10398 | 12247 | 10433 | 45 |
| V4 | 6419 | 1630 | 3395 | 3024 | 0 |
| V67 | 18269 | 4155 | 8417 | 9852 | 0 |
| V8 | 19216 | 6839 | 9285 | 9931 | 0 |
| V9 | 11854 | 8189 | 7957 | 3897 | 0 |
| NP2_1 | | | | | |
| V2 | 2710 | 217 | 1310 | 1400 | 0 |
| V3 | 18361 | 7299 | 10350 | 8011 | 210 |
| V4 | 7114 | 1843 | 3841 | 3273 | 0 |
| V67 | 14377 | 1810 | 6945 | 7432 | 0 |
| V8 | 15519 | 4296 | 7881 | 7638 | 0 |
| V9 | 6513 | 4524 | 4264 | 2249 | 0 |
| NP2_2 | | | | | |
| V2 | 4103 | 263 | 2007 | 2096 | 100 |
| V3 | 22800 | 8172 | 12059 | 10741 | 611 |
| V4 | 11363 | 2889 | 6049 | 5314 | 12 |
| V67 | 23478 | 3881 | 10546 | 12932 | 11 |
| V8 | 28044 | 8740 | 14102 | 13942 | 13 |
| V9 | 8042 | 5730 | 5226 | 2816 | 0 |
| NP2_3 | | | | | |
| V2 | 6140 | 994 | 3838 | 2302 | 78 |
| V3 | 11627 | 3906 | 7001 | 4626 | 120 |
| V4 | 10969 | 3954 | 5022 | 5947 | 0 |
| V67 | 21289 | 3918 | 10186 | 11103 | 17 |
| V8 | 20129 | 6937 | 11029 | 9100 | 0 |
| V9 | 8118 | 5647 | 3703 | 4415 | 0 |
| NP3_1 | | | | | |
| V2 | 7906 | 811 | 4238 | 3668 | 207 |
| V3 | 32105 | 12632 | 17716 | 14389 | 825 |
| V4 | 16218 | 3969 | 8337 | 7881 | 15 |
| V67 | 30354 | 6250 | 14395 | 15959 | 94 |
| V8 | 37768 | 12596 | 19587 | 18181 | 36 |
| V9 | 13252 | 9536 | 7869 | 5383 | 0 |
| NP3_2 | | | | | |
| V2 | 6250 | 497 | 3050 | 3200 | 205 |
| V3 | 29528 | 11272 | 14818 | 14710 | 749 |
| V4 | 13957 | 3149 | 7160 | 6797 | 0 |
| V67 | 28930 | 5124 | 12755 | 16175 | 64 |
| V8 | 34117 | 9790 | 15899 | 18218 | 0 |
| V9 | 11733 | 8323 | 7546 | 4187 | 0 |
| NP3_3 | | | | | |
| V2 | 6727 | 617 | 3509 | 3218 | 162 |
| V3 | 22560 | 7568 | 11919 | 10641 | 487 |
| V4 | 11018 | 2101 | 5513 | 5505 | 11 |
| V67 | 23223 | 3594 | 10978 | 12245 | 64 |
| V8 | 24234 | 6091 | 11329 | 12905 | 0 |
| V9 | 9919 | 6920 | 6052 | 3867 | 0 |
| WR_1 | | | | | |
| V2 | 1648 | 11 | 795 | 853 | 0 |
| V3 | 19022 | 6470 | 9778 | 9244 | 150 |
| V4 | 5424 | 467 | 3226 | 2198 | 0 |
| V67 | 11366 | 1235 | 5178 | 6188 | 0 |
| V8 | 14648 | 2847 | 7299 | 7349 | 0 |
| V9 | 4102 | 2808 | 2967 | 1135 | 0 |
| WR_2 | | | | | |
| V2 | 8785 | 829 | 4615 | 4170 | 105 |
| V3 | 69622 | 31555 | 39481 | 30141 | 1137 |
| V4 | 21264 | 4138 | 11727 | 9537 | 0 |
| V67 | 53554 | 11635 | 23474 | 30080 | 12 |
| V8 | 57501 | 18229 | 30307 | 27194 | 0 |
| V9 | 21938 | 16330 | 13674 | 8264 | 0 |
| WR_3 | | | | | |
| V2 | 2516 | 71 | 1350 | 1350 | 25 |
| V3 | 9845 | 2750 | 5281 | 4564 | 0 |
| V4 | 4828 | 254 | 2498 | 2330 | 0 |
| V67 | 12529 | 1424 | 5985 | 6544 | 0 |
| V8 | 10943 | 1887 | 5690 | 5253 | 0 |
| V9 | 3612 | 2331 | 2079 | 1533 | 0 |

(a)

(b)

Figure S1 : Relative abundances of Bacterial composition at family (a) and genus (b) level in Bulk rock phosphate samples (Bulk_RP) and rhizosphere samples of phosphate native plants Alternanthera caracasana (NP1), Digitaria sanguinalis (NP2), Dittrichia Viscosa (NP3) and wheat crop (WR).

Figure S2 : Rarefaction curves of Shannon index in the studied samples

Figure S3 : heatmap representation of Relative abundances of Bacterial composition at phylum level in Bulk rock phosphate samples (Bulk_RP) and rhizosphere samples of phosphate native plants *Alternanthera caracasana* (NP1), *Digitaria sanguinalis* (NP2), *Dittrichia Viscosa* (NP3) and wheat crop (WR).

Figure S4 : phylogenetic tree of *Actinobacteria* sequences in native plants (NP) samples, bulk phosphate mines (Bulk) samples and wheat rhizosphere (WR) samples. The tree was generated by multiple pairwise alignment of all sequences assigned to *Actinobacteria* derived from Qiime2 output using MEGAX and MAFFT Software. The sequences were then combined in a single queue and aligned by MAFFT. E. coli "NR_024570.1" sequence was set as a reference for the alignment. The phylogenetic tree was constructed by the Maximum likelihood program and a bootstrap analysis was performed with 1000 trials. Three reference actinobacteria strains were used *Actinomyces bovis* NR044862, *Nigerium massiliense* NR173696 and *Actinoplanes consettensis* NR024740. Colors indicated the common or specific clades between the studied samples

Figure S5 : ZymoBIOMICS Microbial Community Standard composition and abundance.
